# Neuroprotective and Neurorestorative Effects of *Holothuria scabra* Extract in the MPTP/MPP^+^-Induced Mouse and Cellular Models of Parkinson’s Disease

**DOI:** 10.3389/fnins.2020.575459

**Published:** 2020-12-21

**Authors:** Kunwadee Noonong, Prasert Sobhon, Morakot Sroyraya, Kulathida Chaithirayanon

**Affiliations:** Department of Anatomy, Faculty of Science, Mahidol University, Bangkok, Thailand

**Keywords:** *Holothuria scabra*, MPP^+^, MPTP, tyrosine hydroxylase, α-synuclein, striatal dopamine

## Abstract

Extracts from *Holothuria scabra* (HS) have been shown to possess anti-inflammation, anti-oxidant and anti-cancer activities. More recently, it was shown to have neuroprotective potential in *Caenorhabditis elegans* PD model. Here, we assessed whether HS has neuroprotective and neurorestorative effects on dopaminergic neurons in both mouse and cellular models of PD. We found that both pre-treatment and post-treatment with HS improved motor deficits in PD mouse model induced with 1-methyl-4-phenyl-1,2,3,6-tetrahydropyridine (MPTP) as determined by grid walk test. This was likely mediated by HS protective and restorative effects on maintaining the numbers of dopaminergic neurons and fibers in both substantia nigra pars compacta (SNpc) and striatum. In a cellular model of PD, HS significantly attenuated 1-methyl-4-phenylpyridinium (MPP^+^)-induced apoptosis of DAergic-like neurons differentiated from SH-SY5Y cells by enhancing the expression of Bcl-2, suppressing the expression of cleaved Caspase 3 and preventing depolarization of mitochondrial membrane. In addition, HS could stimulate the expression of tyrosine hydroxylase (TH) and suppressed the formation of α-synuclein protein. Taken together, our *in vivo* and *in vitro* findings suggested that HS is an attractive candidate for the neuroprotection rather than neurorestoration in PD.

## Introduction

Parkinson’s disease (PD) is an age-related neuro-degenerative disorder of the central nervous system that affects more than 10 million people worldwide (Faisal Nouroz et al., 2014). PD is characterized by the presence of intracytoplasmic inclusion Lewy bodies (LB) in nigral dopaminergic neurons, followed by cell death (Silvia Mandel et al., 2009). Although, PD has been discovered many decades ago, the cause of PD is still unclear, and there is no complete cure yet for Parkinson’s disease. Levodopa (L-dopa), dopamine precursor, provides only symptomatic relief but fails to restore neuronal degeneration (Faisal Nouroz et al., 2014). Besides, there is no proven therapy that could prevent neuronal death, i.e., neuroprotection, or restore sick neurons to a normal state, i.e., neurorestoration (Dawson and Dawson, 2002). Interestingly, several recent studies indicate that natural products have potentials as alternative treatments that can confer both neuroprotection and neurorestoration. Phytochemicals from medicinal plants such as triterpene, polyphenol, flavonoid, alkaloid, and glycosides have been reported to have neuroprotective effects against stroke, Alzheimer’s disease, Parkinson’s disease, and Huntington’s disease (Sun et al., 2015; Solayman et al., 2017), while resveratrol, curcumin, and ginsenoside were demonstrated to possess an anti-PD effect (Pangestuti and Arifin, 2017). Additionally, many marine organisms also contain biologically active components with similar therapeutic properties. These natural products could be developed into nutraceuticals for neuroprotection and neurorestoration (Bordbar et al., 2011; Pangestuti and Kim, 2011). Sea cucumbers have been reported to possess these potentials, and they have been used in traditional Chinese medicine as tonics and delicacies for a long time. Among these marine organisms is the white sea cucumber, *Holothuria scabra* (HS), which is an echinoderm belonging to phylum Echinodermata, class Holothuroidea. They are abundant and widely distributed in Africa, Red Sea, South China Sea, South-Pacific, South East Asia, and the Indian Ocean. HS is rich in triterpene glycosides, such as scabraside A, scabraside B, scabraside D, holothurin A3, holothurin A4, fuscocineroside and 24-dehydroechinoside A, phenols, and flavonoids (Honey-Escandón et al., 2015; Pangestuti and Arifin, 2017; Khotimchenko, 2018). In addition, phenolic compounds including friedelin, 3-hydroxybenzaldehyde, and 4-hydroxybenzaldehyde isolated from the whole body of HS showed a high antioxidant activity (Nobsathian et al., 2017). In this context, 4-hydroxybenzaldehyde displayed a significant inhibition of superoxide anion generation and elastase release by human neutrophils (Kuo et al., 2017). Moreover, members of our group have recently reported that HS extracts exhibited anti-Parkinson potential in *Caenorhabditis elegans* model (Chalorak et al., 2018). However, the effects and mechanisms of HS chemicals on neuroprotection and/or neurorestoration against PD in a mammalian model remains to be demonstrated and elucidated. Therefore, in this study, we aimed to evaluate the effects of HS in the prevention and restoration of PD in MPTP/MPP^+^-induced mouse and cellular models of Parkinson’s disease.

## Materials and Methods

### Chemicals and Reagents

Culture medium DMEM/F12 was purchased from FUJIFILM Wako Pure Chemical Corporation (Osaka, Japan). Fetal bovine serum, penicillin–streptomycin, and phosphate buffer solution were purchased from Invitrogen corporation (Massachusetts, United States). 3-(4,5-Dimethylthiazol-2-yl)-2,5-diphenyl tetrazolium bromide (MTT), retinoic acid (RA),12-O-tetradecanoylphorbol-13-acetate (TPA), and PMSF protease inhibitor were purchased from Sigma-Aldrich Corporation (St. Louis, MO, United States). Hoechst 33258 was purchased from Abcam (Cambridge, United Kingdom). RIPA buffer, anti-Bcl-2 (15071S), anti-Bax (2774S), and anti-β-actin (4697S) were purchased from Cell Signal Technology (Massachusetts, United States). Anti-cleaved caspase 3 (ab184787), anti-tyrosine hydroxylase (ab75875), anti-α-synuclein (ab3309), and JC-1 assay kit (ab113850) were purchased from Abcam (Cambridge, United Kingdom). Secondary antibody HRP-conjugated anti-rabbit IgG and anti-mouse IgG were purchased from SouthernBiotech (Birmingham, United States). All other chemicals used were of analytical grade.

### Animals

Wild-type C57BL/6 male mice, 7–8 weeks old (young adult) weighing 20–25 g, were used in this experiment. The animals were randomly housed (*n* = 5 per cage) for 7 days to acclimatize under a normal 12/12 h light/dark. They were housed in an ambient temperature of 23 ± 1°C with relative humidity of 60 ± 10%. Food and water were provided *ad libitum*. Fully mature *Holothuria scabra* (HS) weighing between 350 and 500 g were collected from the Andaman sea and maintained at the Coastal Fisheries Research and Development Center, Prachuapkhirikhun, Thailand. They were kept in concrete tanks containing filtered sea water with continuous aeration and fed with algal-based feed until used for the experiments. All protocols of animal handling and experiments in this study were approved by Animal Care and Use Committee, Faculty of Science, Mahidol University.

### Preparation of HS Extract

HS were anesthetized on ice, and their body walls were dissected out, cut into small pieces, immersed in liquid nitrogen, pulverized, macerated in 95% ethanol solution for 7 days, then filtered twice. The ethanol phase was evaporated under vacuum, then the powder was kept at −20°C until use. The compounds in HS were characterized by using nuclear magnetic resonance spectroscopy as previously described (Nobsathian et al., 2017).

### HS Administration

**FIGURE 1:**
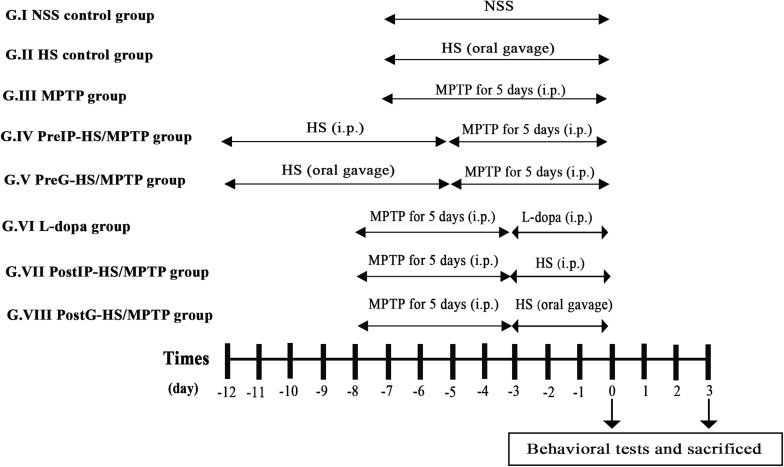
Timeline of th eexperimental procedure. Mice were randomly assigned to eight groups (*n* = 20 per group) consisting of two control [normal saline solution (NSS) control group and *Holothuria scabra* (HS) control group] and six study groups. The mice groups III to VIII were managed to subsequent behavioral tests after completed with 1-methyl-4-phenyl-1,2,3,6-tetrahydropyridine (MPTP) treatment at day 0 and day 3. HS extract was dissolved in NSS (0.9% NaCl). Mice were euthanized by cervical dislocation.

To investigate whether HS possesses neuroprotective and neurorestorative potentials, we examined the effects of pretreatment and post-treatment with HS in MPTP-treated C57BL/6 mouse model of PD. Mice were randomly assigned to eight groups (*n* = 20 per group) consisting of two control and six experimental groups. The mice groups III to VIII were managed to subsequent behavioral tests after completed with MPTP treatment at day 0 and day 3 (Figure 1). HS extract was dissolved in normal saline solution (NSS, 0.9% NaCl).

Group I (NSS control group): mice were intraperitoneally injected with normal saline solution for 7 days.

Group II (HS control group): mice were orally administered with HS 10 mg/kg daily for 7 days.

Group III (MPTP group): mice received intraperitoneal injection of MPTP alone at a dose of 25 mg/kg/day for 5 consecutive days.

Group IV (PreIP-HS/MPTP group): mice were intraperitoneally treated with HS (10 mg/kg/day) for 7 consecutive days and then MPTP (25 mg/kg/day) for 5 consecutive days.

Group V (PreG-HS/MPTP group): mice were orally administered with HS (200 mg/kg/day) for 7 consecutive days and intraperitoneal injection of MPTP (25 mg/kg/day) for 5 consecutive days.

Group VI (L-dopa group): mice were intraperitoneally injected with MPTP (25 mg/kg/day) for 5 consecutive days and followed by L-dopa (10 mg/kg/day) for 3 consecutive days.

Group VII (PostIP-HS/MPTP group): mice were intraperitoneally injected with MPTP (25 mg/kg/day) for 5 consecutive days. After that, mice were intraperitoneally administered with HS (10 mg/kg/day) for 3 consecutive days.

Group VIII (PostG-HS/MPTP group): mice received intraperitoneal injection of MPTP (25 mg/kg/day) for 5 consecutive days and followed by oral administration of HS (200 mg/kg/day) for 3 consecutive days.

All mice received training trials with an intertrial interval of 4–5 min for 4 days before experiment.

### Grid Walk Test

The grid walk test was used to determine motor impairments of limb functioning and placement deficits during locomotion. Mice were placed on a grid (40 × 60 × 100 cm; L × W × H with 2.5 × 2.5 cm grid spaces). The number of paw slips through the grid were recorded.

### Immunohistochemistry

Mice were euthanized by cervical dislocation. To obtain anterior and posterior parts of the brain, mouse brains were carefully removed and cut in coronal plane between bregma 0 and −2.12 mm. Right and left hemispheres were then separated along the sagittal plane. Then, the brains were immediately fixed in Bouin’s solution for 24 h and later embedded in paraffin blocks. Serial sections of mouse brains (5 μm) were deparaffinized and rehydrated. Endogenous peroxidase activity in the tissue was reduced by treatment with 3% H_2_O_2_ in 100% methanol. Then, the sections were incubated for 2 h in blocking reagent (4% normal goat serum in 0.1 M PBST pH 7.4). The sections were incubated for 2 h with polyclonal antibody against tyrosine hydroxylase (1:1,000). Subsequently, the sections were washed and then incubated with secondary antibody, HRP-conjugated goat anti-rabbit IgG (1:4,000) for 1 h. Peroxidase activity was visualized by reacting with DAB in 0.05 M tris-buffered saline (pH 7.6). The stained tissues were observed and photographed under Nikon E600 microscope equipped with Nikon digital DXM1200 camera, using an ACT-1 software package. Tyrosine hydroxylase (TH) intensity in the SNpc and striatum was measured in TH-positive cells and fibers, and quantified with image J version 1.51k.

### Cells and Cell Culture

SH-SY5Y cells were obtained from the American Type Culture Collection (United States) and cultured in DMEM/F12 with 10% fetal bovine serum (FBS), penicillin (100 U/ml), and streptomycin (100 μg/m). Culture medium was changed every 3–4 days. Cells were maintained in a humidified 5 CO_2_ atmosphere at 37°C. To initiate neurodifferentiation, SH-SY5Y cells were sequentially treated with 10 μM of retinoic acid (RA) for 3 days followed with 80 ηM of 12-O-tetradecanoylphorbol-13-acetate (TPA) for 3 days. Finally, SH-SY5Y cells were differentiated and developed into dopaminergic (DAergic)-like neurons as shown by dopamine (DA) synthesis and dopamine transporter (DAT) expression (Xie et al., 2010).

### Cytotoxicity Assay for *Holothuria scabra*

DAnergic-like neurons were plated into 96-well plates at a density of 5,000 cells/well. After 24 h, cells were treated with HS for 24 and 48 h in concentrations ranging from 0.5, 1, 5, 10, and 50 μg/ml. The control group was treated with 0.1% DMSO. After treatment, cell viability was determined by using the MTT assay. Briefly, 100 μl of MTT at a concentration 0.5 mg/ml was added into each well of a 96-well plate. After 3 h of incubation at 37°C, MTT solution was removed, and then formazan crystals were dissolved with 100 μl of DMSO. The purple color being formed was measured spectrophotometrically at 570 nm (with reference OD 690). Cell viability was calculated using Prism 6 software. The suitable concentration of HS was selected for a later experiment.

### Cell Treatments With *Holothuria scabra* and 1-Methyl-4-Phenylpyridinium

HS extract was dissolved in DMEM/F12 with 2% FBS. The HS concentrations at 0.5, 1, and 5 μg/ml were added to DAnergic-like neurons. Then, cells were maintained in a humidified 5% CO_2_ atmosphere at 37°C for 24 and 48 h. After washing with 1 × PBS, 500 μM of MPP^+^ was added into the culture plate and incubated for 24 h in a humidified 5% CO_2_ atmosphere at 37°C. Ultimately, cell viability was determined by using the MTT assay.

### Hoechst 33258 Staining

Hoechst 33258 staining was used to illustrate DNA fragmentation condensation that occurred in apoptotic cells. DAnergic-like neurons at 1.5 × 10^5^ was cultured on a cover slip and then fixed with 4% formaldehyde for 10 min. Subsequently, fixative solution was removed, and drops of Hoechst stain, at a concentration 2 μg/ml, were added onto the cover slip. Cells were incubated at room temperature for 15 min. After that, cells were washed with 1 × PBS, and cells with nuclear chromatin condensation were observed and recorded with Olympus Live Cell Fluorescence Imaging System (IX-83ZDC) with excitation at 352 nm and 20× magnification.

### Mitochondria Membrane Potential (ΔΨm)

The mitochondria membrane potential (MMP) is depolarized when a cell undergoes apoptosis. Thus, MMP is an essential parameter indicating mitochondrial function, and it can be measured using the JC-1 dye. Briefly, DAergic-like neurons were seeded in 96-black well plates and treated with HS as described above. Cells were washed with PBS and then incubated with JC-1 staining solution (10 μM) in a dark room for 10 min at 37°C and 5% CO_2_. After washing with PBS, red fluorescence intensity (aggregate) was measured at Ex/Em: 535/590 nm, and green fluorescence intensity (monomer) was measured at Ex/Em: 490/530 nm. The ratio between red/green was measured by dual emission florescence microplate reader (Tecan Spark 10M, Bioexpress, Switzerland). In addition, JC-1 staining was observed by dual emission fluorescence microscopy (Olympus Live Cell Fluorescence Imaging System, IX-83ZDC).

### Western Blot Assay

Cells were lysed on ice with 1 × RIPA buffer containing PMSF protease inhibitor. Cell lysates were obtained by centrifugation at 10,000 RPM for 10 min at 4°C. Protein concentrations were estimated by BCA assay. Thirty micrograms of proteins from each lysate were electrophoresed and then transferred to nitrocellulose membranes. After blocking for 60 min in TBST containing 5% non-fat dried milk, the membranes were incubated overnight at 4°C with primary antibodies against Bcl-2 (1:700), Bax (1:700), β-actin (1:1,000), TH (1:1,000), and α-synuclein (1:1,000) followed by incubation with HRP-conjugated anti-rabbit or anti-mouse IgG at room temperature for 2 h. Protein bands were observed by enhanced chemiluminescence ECL on radiographic films. Relative quantities of proteins of interest were quantified by densitometric scanning and analyzed by Image J.

### Statistical Analysis

Results were represented as means ± SEM and analyzed by using GraphPad Prism 5.0 (GraphPad Software). Statistical comparisons were determined by one-way ANOVA, followed by two-tailed Student’s *t*-test to determine significant differences between the two groups. *P*-values of less than 0.05 were considered statistically significant.

## Results

### *Holothuria scabra* Improved Behavioral Performances of 1-Methyl-4-Phenyl-1,2,3,6-Tetrahydropyridine-Induced Parkinson’s Disease Mice

**FIGURE 2:**
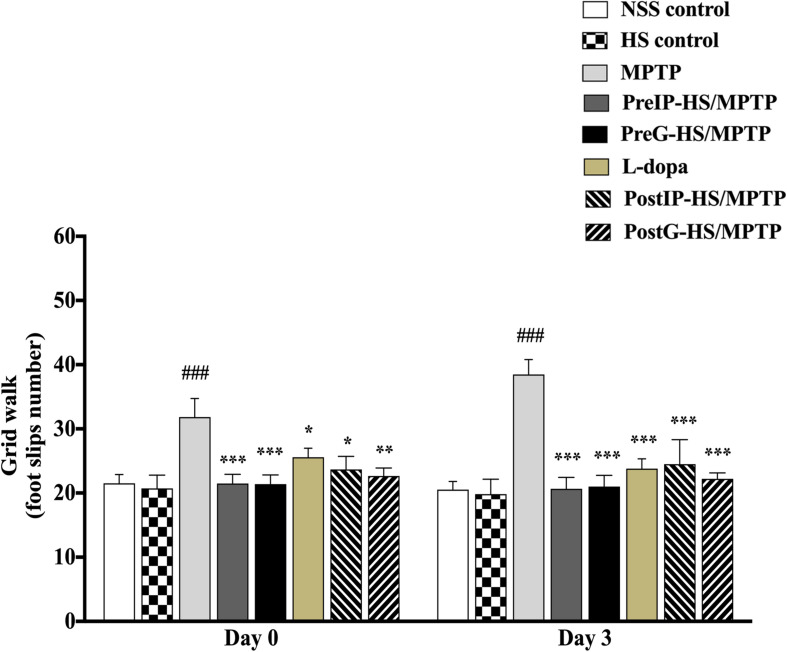
The number of foot slips during grid walk on day 0 and 3 after MPTP administration is shown in histograms. Data are presented as the means ± standard error of the mean (SEM) *P < 0.05, **P < 0.01, and ***P < 0.001, in comparison with the NSS-injected group. ^###^*P* < 0.001, in comparison with the MPTP-injected group.

Motor impairment is typically related to dopaminergic nerve injury in PD. Thus, to examine potential neuroprotective/neurorestorative effect of HS on motor impairment, we investigated the motor coordination activity and locomotor activity of mice using grid walk test. When mice were challenged with MPTP, their foot slip number during grid walk was significantly increased in association with impairments of limb motor functions and placement deficits during locomotion. On day 0, mice injected with MPTP exhibited higher foot slip number during grid walk (31.8 ± 2.90 s), compared with the control (23.7 ± 2.04 s) (Figure 2). We examined neuroprotective and neurorestorative effects of HS on motor impairments using pretreatment and post-treatment, respectively (see section “Material and Methods” for group designation). Interestingly, the PreIP-HS/MPTP and PreG-HS/MPTP groups showed significantly reduced foot slip number during grid walk (21.5 ± 1.41 and 21.4 ± 1.42, respectively). In addition, there was a significant reduction of motor impairment in foot slip number in L-dopa, PostIP-HS/MPTP, and PostG-HS/MPTP groups when compared with the MPTP group (Figure 2). On day 3, all experimental groups showed significant reduction of foot slip number when compared to the MPTP group (Figure 2). The results of these behavioral tests indicated that HS showed efficacy in prevention and restoration of motor function in MPTP-treated mice.

### *Holothuria scabra* Could Protect and Restore Dopamine Neurons and Fibers in Substantia Nigra Pars Compacta and Striatum of 1-Methyl-4-Phenyl-1,2,3,6-Tetrahydropyridine-Induced Parkinson’s Disease Mice

**FIGURE 3:**
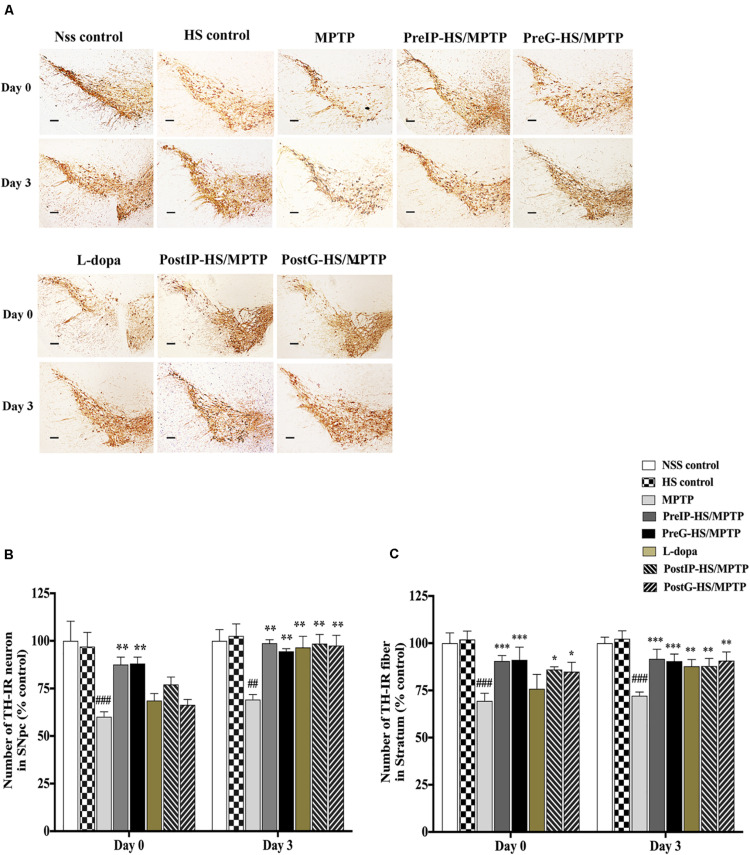
**(A)** Dopaminergic neurons and fibers were detected with tyrosine hydroxylase (TH) immunostaining in the substantia nigra pars compacta (SNpc) and striatum of mice brain at high magnification (20 ×) containing immunolabeled TH-positive dopamine (DA) neurons and fibers (brown). **(B)** Number of dopaminergic neurons with TH immunostaining in SNpc on days 0 and 3. **(C)** Number of dopaminergic fibers with TH immunostaining in the striatum. Data are presented as the means ± standard error of the mean (SEM) *P < 0.05, **P < 0.01, and ***P < 0.001, in comparison with the NSS-injected group. ^##^*P* < 0.01, and ^###^*P* < 0.001, in comparison with the MPTP-injected group.

To further assess the neuroprotective and neurorestorative effects of HS at the cellular level, weexamined the expression of tyrosine hydroxylase in the pars compacta of the substantia nigra (SNpc) and striatum (STA) of mice by immunohistochemistry and counted the numbers of positive cells and fibers that appeared reddish brown in contrast to the background (Figure 3A). At day 0 in the SNpc and STA of mice treated with MPTP, the numbers of TH-positive cells and fibers significantly decreased to 60.1 ± 4.59 and 69.4 ± 19.34% (Figures 3B,C) when compared to the untreated control (100 ± 20.6 and 100 ± 23.6%). By contrast, the PreIP-HS/MPTP group showed significant increases in both TH-positive cells in SNpc (87.6 ± 8.75%) and TH-positive fibers in STA (90.6 ± 13.22%) when compared to the MPTP group. Similarly, the PreG-HS/MPTP group also showed a significant increase in the TH-positive cells in the SNpc (88.1 ± 15.84%) and TH-positive fibers in the STA (91.2 ± 15.94%) when compared with the MPTP group (Figures 3B,C). The L-dopa group did not exhibit a significant increase in both TH-positive cell in the SNpc and TH-positive fibers in STA when compared to the MPTP group. Both PostIP-HS/MPTP and PostG-HS/MPTP did not show significant increase in positive neurons in the SNpc when compared with the MPTP group, but both groups showed a significant increase in positive fibers in the STA (Figures 3B,C). On day 3, the numbers of both TH-positive cells and TH-positive fibers increased in PostIP-HS/MPTP, PostG-HS/MPTP, and L-dopa group when compared with the MPTP group, and they reached the level slightly lower than those of the PreIP-HS/MPTP and PreG-HS/MPTP groups (Figures 3B,C). This implied that post-treatment with HS after MPTP-induced PD could partially restore DA neurons and fibers in the SNpc and STA. Taken together, similar to behavioral improvement, it appeared that HS provided a higher degree of neuroprotection than neurorestoration for both DA neurons and fibers.

### Cytotoxicity Assay

**FIGURE 4:**
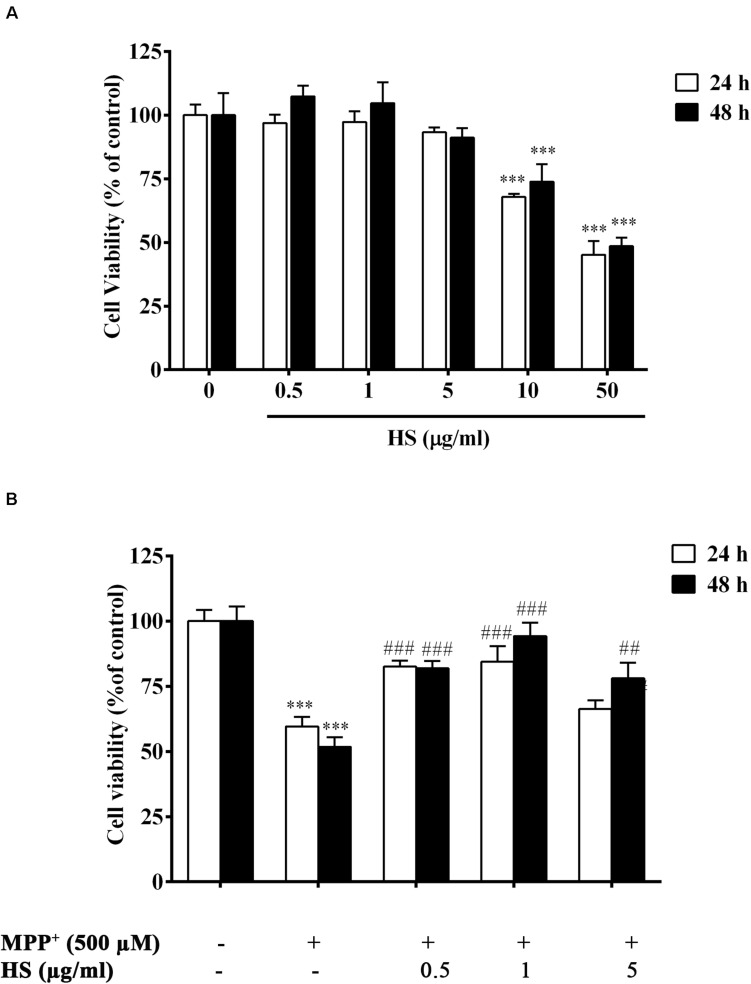
**(A)** Viability of DAergic-like neurons after incubation with HS at concentrations of 0.5, 1, 5, 10, and 50 μg/ml for 24 and 48 h. **(B)** Effects of HS extracts on 1-methyl-4-phenylpyridinium (MPP^+^)-induced toxicity in DAergic-like neurons. DAergic-like neurons were pretreated with HS (0.5, 1, and 5 μg/ml) for 24 and 48 h and then exposed to 500 μM MPP^+^ for 24 h. Cell viability was determined by 3-(4,5-dimethylthiazol-2-yl)-2,5-diphenyl tetrazolium bromide (MTT). Data are presented as the means ± standard error of the mean (SEM) and ^∗∗∗^*P* < 0.001, in comparison with the untreated group. ^##^*P* < 0.01, and ^###^*P* < 0.001, in comparison with the MPP^+^-treated group.

Cytotoxicity assay was used to assess the possible toxicity of HS on DAergic-like neurons. When DAergic-like neurons were incubated with HS at concentrations of 0.5, 1, 5, 10, and 50 μg/ml for 24 h, cell viability was significantly decreased only at high concentrations of 10 and 50 μg/ml to 67.8 ± 1.21 and 45.2 ± 5.38%, respectively. When the incubation time was extended to 48 h, similar results were obtained (Figure 4A). This indicated that HS was not toxic to DAergic-like neurons at low concentrations ranging from 0.5 to 5 μg/ml. Therefore, these concentrations (0.5, 1, and 5 μg/ml) of HS were used for further *in vitro* experiments.

### *Holothuria scabra* Extract Reduced Death of the 1-Methyl-4-Phenylpyridinium-Treated Dopaminergic-Like Neurons

DAergic-like neurons were pretreated with HS extract at 0.5, 1, and 5 μg/ml for 24 and 48 h and then were exposed to 500 μM MPP^+^ for 24 h. Cell viability was determined by MTT assay as described in the section “Materials and Methods.” The results showed a significant decrease in the viability of the DAergic-like neurons following exposure to 500 μM MPP^+^ for 24 h (59 ± 5.1%). Pretreatments with HS at all doses showed a significant increase in cell viability when compared to only MPP^+^ treatment with maximal number of viable cells at a concentration of 1 μg/ml of HS and 48 h incubation (Figure 4B). This indicated the neuroprotective ability of HS against MPP^+^-induced cell death in a time-dependent manner.

### *Holothuria scabra* Extracts Reduced Mitochondria Membrane Potential (ΔΨm) Against 1-Methyl-4-Phenylpyridinium-Induced Dopaminergic-Like Neurons

**FIGURE 5:**
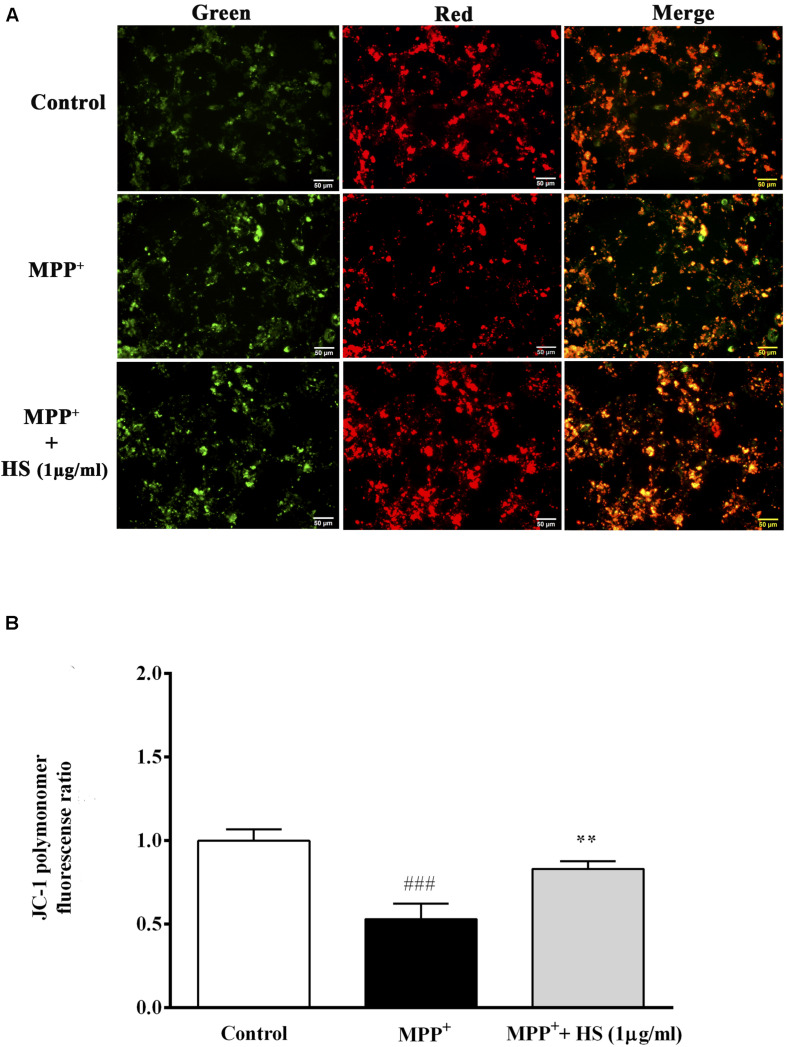
The mitochondria membrane potential (MMP) was assessed by JC-1 staining. Green JC-1 monomers indicate depolarized MMP, while red JC-1 dimer indicates normal MMP. Merged panel represents colocalization between red dimer and green monomer fluorescence. **(A)** JC-1 staining in untreated DAergic-like neurons, DAergic-like neurons treated with 500 μM MPP^+^, DAergic-like neurons with 1 μg/ml of HS pretreatment, followed by 500 μM MPP^+^. **(B)** Quantitative analysis of relative fluorescence between red and green in each group as measured by microplate reader. Data are presented as the means ± standard error of the mean (SEM) **P < 0.01, in comparison with the untreated group. ^###^*P* < 0.001, in comparison with the MPP^+^-treated group.

Stable mitochondrial membrane potential (MMP) provided a valuable indicator of a heathy cell. The changes in MMP is considered to be one of the important events related to apoptosis (Dhanalakshmi et al., 2015). Amelioration of mitochondrial dysfunction has been proposed as a neuroprotective effect in neurodegeneration (Zhong et al., 2018). MMP changes were determined by JC-1, a mitochondria-specific dye. In normal MMP, JC-1 aggregated and shows red fluorescence, while depolarized MMP shows green fluorescence, representing apoptotic cells (Sivandzade et al., 2019). DAergic-like neurons exposed to 500 μM MPP^+^ for 24 h showed significant depolarization of MMP as indicated by the lower ratio of red-to-green florescence when compared to the control group. Also, in Figure 5A, the merged picture revealed high green fluorescence intensity in the MPP^+^-treated group, representing unhealthy cells undergoing apoptosis. By contrast, pretreatment with HS at 1 μg/ml remarkably increased red fluorescence, while there was a slightly decreased green fluorescence in DAergic-like neurons (Figure 5B). These results indicated that treatments with HS could attenuate the depolarization of MMP by MPP + in DAergic-like neurons.

### *Holothuria scabra* Extracts Attenuated 1-Methyl-4-Phenylpyridinium -Induced Apoptosis and Cleaved Caspase 3 Protein Expression

**FIGURE 6:**
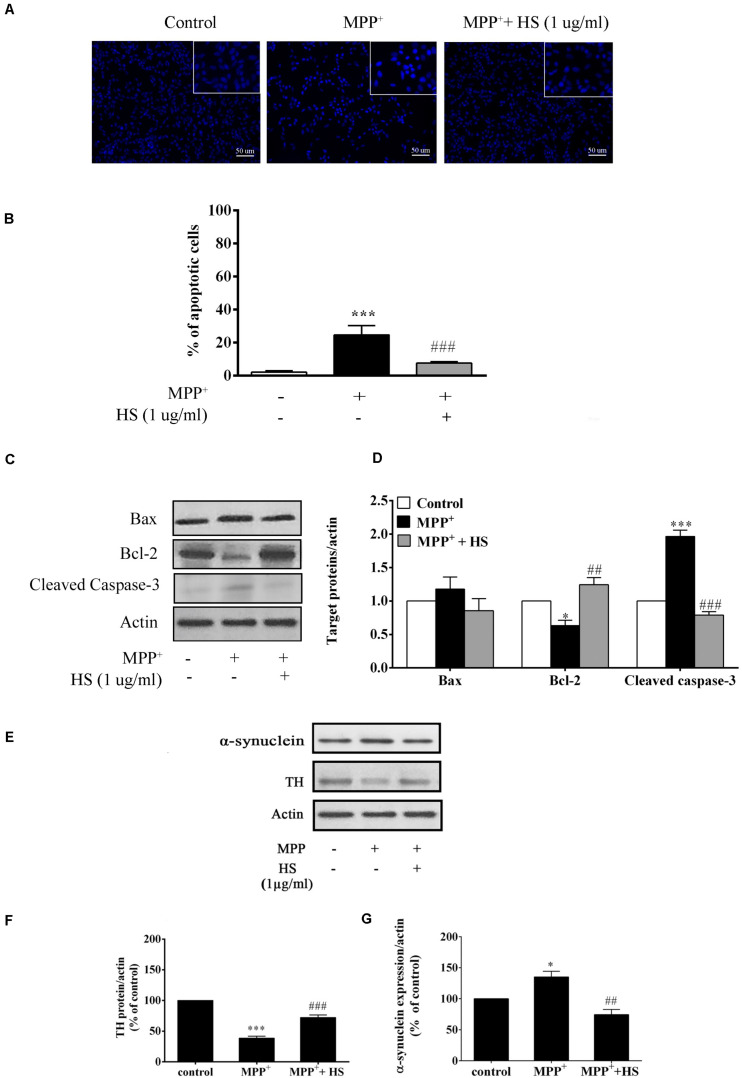
**(A)** Effects of HS on MPP^+^-induced DAergic-like neuron apoptosis shown by Hoechst 33258 stain; upper panel indicates DNA condensation in the nucleus. **(B)** Histograms showed percentage of apoptotic cells. **(C)** Expression levels of Bax, Bcl-2, and cleaved caspase 3 protein as determined by Western blot (analysis; actin was used as the control and for normalizing the intensities of the target proteins in untreated, treated with 500 μM MPP^+^, or pretreated with 1 μg/ml of HS then followed by 500 μM MPP^+^. **(D)** Histograms showed the relative intensity of Bax, Bcl-2, and cleaved caspase 3 expressions as normalized by actin. **(E)** Expression levels of tyrosine hydroxylase (TH) and α-synuclein in MPP^+^-induced DAergic-like neurons by Western blot, comparing between the untreated control, treated with 500 μM MPP^+^, treated with 1 μg/ml of HS and 500 μM MPP^+^. **(F)** Histograms showed the relative intensity of TH expression as normalized by action. **(G)** Histograms showed the relative intensity of α-synuclein expression as normalized by action. Data are presented as the means ± standard error of the mean (SEM) ^∗^*P* < 0.05, and ^∗∗∗^*P* < 0.001, in comparison with the untreated group. ^##^*P* < 0.01, and ^###^*P* < 0.001, in comparison with the MPP^+^-treated group.)

To determine whether HS protected DAergic-like neurons from MPP^+^-induced apoptosis, Hoechst staining and Western blot analysis were conducted to analyze the nuclear morphology and apoptotic protein expressions. The results revealed that exposure to MPP^+^ significantly increased the number of apoptotic cells with DNA condensation (24.6%) compared to the control group (2.1%). The number of apoptotic cells significantly decreased to 7.6% when cells were pretreated with 1 μg/ml of HS (Figures 6A,B). To clarify the role of HS in attenuating MPP^+^-induced apoptosis, expressions of Bax, Bcl-2, and cleaved caspase 3 were investigated by Western blot. The results demonstrated that exposure to MPP^+^ significantly increased the level of cleaved caspase 3 expression, while it decreased Bcl-2 expression with a slight, but non-significant, increase in the level of Bax (Figures 6C,D). Pretreatments with HS significantly caused a significant increase in Bcl-2 and a reduction in cleaved caspase 3 expression, while Bax expression was also decreased but at a level not significant from that of the MPP^+^-treated cells (Figures 6C,D). This finding supported the notion that HS extract attenuated MPP^+^-induced apoptosis by increasing the expression of anti-apoptotic protein (Bcl-2) while suppressing the expression of apoptotic proteins, especially cleaved caspase 3.

### *Holothuria scabra* Extract Increased Tyrosine Hydroxylase and Decreased α-Synuclein Expressions

To determine the neuroprotective effect of HS against MPP^+^-treated cells, the expression of tyrosine hydroxylase (TH), a neuron-specific marker for DAergic neurons, was determined by Western blot. After 24 h of MPP^+^ treatment, the expression of TH protein in treated cells was reduced by 63.8% compared with that in untreated cells. Cells pretreated with HS showed significantly increased expression of TH protein by 29.6% compared with cells treated with MPP alone (Figures 6E,F). Furthermore, cells pretreated with HS showed significantly decreased level of α-synuclein by 65.4% compared with cells treated with MPP^+^ alone (Figures 6E,G). These results indicated that pretreatment with HS promoted TH synthesis while suppressing the formation of α-synuclein protein.

## Discussion

MPTP-induced mouse model of Parkinson’s disease has been widely used and accepted for studying molecular mechanisms correlated with behavioral changes (Dawson and Dawson, 2003; Meredith and Rademacher, 2011) and initial screening of new treatments (Perry et al., 1985; Tatton and Greenwood, 1991; Meredith and Rademacher, 2011). In this context, the C57BL/6 mouse strain that is most sensitive to MPTP has been employed to study pre- and post-treatment (Przedborski et al., 2000; Sedelis et al., 2000; Bhaduri et al., 2018). The dosage and time of MPTP regimen had been applied as a subacute MPTP mouse PD model (Serra et al., 2008; Shi et al., 2016). The results showed that MPTP administration to mice induced the impairments of limb motor function and placement during locomotion, concomitantly with a significant loss of TH-positive cells and their fibers in the SNpc and in the striatum. Interestingly, HS pretreatment via intraperitoneal and oral administrations could improve the limb placement deficit without any toxicity (Supplementary Figures S1A,B). Additionally, we too observed the corresponding significant increases in DA neurons and fibers in the SNpc and STA in the pretreatment groups (Groups IV and V). As expected, these data supported the notion that HS could protect loss of dopaminergic cells with their fibers in an MPTP-induced PD mouse model, leading to behavioral improvement.

In addition to its neuroprotective effect, we further asked whether HS could also provide neurorestoration, a condition in which damaged dopaminergic neurons that still survived could be restored to their normal state (Dawson and Dawson, 2002; Airavaara et al., 2012; Francardo et al., 2017). To this end, we found that mice receiving post-treatments with HS as well as treatment with levodopa showed significant decreases in the motor impairments on day 0 and day 3 when compared with mice treated with MPTP alone. However, the degree of improvement was slightly less than the pretreated mice. L-dopa, known as a dopamine replacement, is most effective in PD. This drug can improve the motor disturbances associated with a loss of nigral dopaminergic neurons. Additionally, the restored TH-positive neurons and their fibers in L-dopa group and both post-treatment of HS performed by immunohistochemical detection were positively presented on day 3, but not on day 0. In this regard, Jin Young Shin and Hyun-Jung Park et al. demonstrated that L-dopa could rescue DA neuron death through the induction of cell survival and the suppression of apoptotic signaling in MPTP-treated PD mice via ERK activation (Shin et al., 2009). Furthermore, increased survival of dopaminergic neurons by L-dopa was dramatically observed by immunohistochemical detection. Therefore, L-dopa should be a positive control in this experiment. Similar with our neurorestoration investigation, the extract from *Holothuria scabra* enriched with triterpene glycosides and polyphenol contents could restore DA neuron after 6-OHDA treatment in *Caenorhabditis elegans* model of PD (Chalorak et al., 2018). Taken together, HS exerted both neuroprotective and neurorestorative effects against deficits caused by MPTP-induced PD in mouse model, and that HS has more neuroprotective than neurorestorative efficacy.

With the neuroprotective effectiveness of HS in the mice model, we performed further investigations using MPP^+^-induced DAergic-like neurons as a cellular model of PD to understand the underlying mechanisms of neuroprotection of HS. Herein, SH-SY5Y cells were differentiated by sequential treatments with RA and TPA to become mature DAergic-like neurons as performed in many studies (Xie et al., 2010; Xicoy et al., 2017). To ensure that HS did not have any undesirable cytotoxicity on DAergic-like neurons, we performed MTT assay (Xicoy et al., 2017) and found that HS was not cytotoxic to DAergic-like neurons at concentrations below 5 μg/ml. Likewise, a previous investigation of bioactive saponin in astragaloside IV up to 50 μM did not exert any significant toxicity in SH-SY5Y cells as measured by the MTT assay (Zhang et al., 2012). Resveratrol (5 μM), a natural polyphenolic compound, slightly promotes SH-SY5Y cell proliferation (Lee et al., 2007). Experimental evidences indicated that many natural products derived from plants, including flavonoids, steroidal lactones, alkaloids, saponins, etc., exhibited a neuroprotective potential against Parkinson’s disease (Subash et al., 2014). Meanwhile, the main bioactive compounds in HS comprise terpene, flavonoid, polyphenol, and alkaloid groups (Sara Bordbar et al., 2011), which might not be overly toxic to SY5Y cells. Also, these chemicals have multifunctional properties, especially in anti-oxidation property. Consequently, we conducted a pretreatment experiment and found that HS could prevent DAergic-like neuron death from MPP^+^ exposure with maximum protection at 1 μg/ml. These findings are similar to recent reports involving neuroprotection activities of bioactive compounds of plant and marine products. For instances, pretreatment with curcumin at 1 μM could protect SH-SY5Y cells from MPP^+^-induced cell death (Yu et al., 2010). Pretreatment with 20 μM of resveratrol could also protect SH-SY5Y cells from rotenone-induced neurotoxicity (Lin et al., 2014). Recently, it has been suggested that exposure to MPP^+^ resulted in the death of the DAergic-like neuron due to inhibition of the complex I in mitochondrial electron transport chain, and subsequent increase in ROS generation, leading to mitochondria membrane disruption and ATP reduction (Keane et al., 2011). Furthermore, the depolarized mitochondria membrane and the permeabilization of the outer membrane can facilitate the release of cytochrome C to promote the apoptosis pathway, causing DNA damage and cell death (Jackson-Lewis and Smeyne, 2005; Keane et al., 2011). In fact, PD is a multifactorial disease caused by oxidative stress and mitochondrial dysfunctions. Drugs that could attenuate mitochondrial impairment and render neuroprotection have gained considerable interest in PD treatment (Thomas and Beal, 2010; Jin et al., 2014; Luo et al., 2015). In our findings, pretreatment of these DAergic-like neurons with 1 μg/ml of HS could decrease mitochondria depolarization, thus resulting in the reduced expression of proapoptosis protein (BAX) and cleaved Caspase 3 expressions and the increased expression of anti-apoptosis protein (Bcl-2) when compared with MPP^+^ alone. This intriguing link seems mediated by the neuroprotective effect of HS on the MPP^+^-induced mitochondria dysfunction leading to apoptosis. Beside its negative effect on mitochondria dysfunction, several *in vitro* studies showed that MPP^+^ can induce α-synuclein expression and aggregation (Cai et al., 2009), which resulted from the impairment of the ubiquitin–proteasome system (UPS) and autophagy–lysosome pathway (ALP) (Ganguly et al., 2018). It has been proposed that α-synuclein plays an important role in initiated neurodegeneration in PD because it can interact with active biomolecules causing mitochondrial impairment, endoplasmic reticulum stress, and synaptic dysfunction (Longhena et al., 2017; Ghiglieri et al., 2018). Agents that can stabilize, degrade, clear, or inhibit the aggregated α-synuclein have been proposed as therapeutic drugs for PD (Javed et al., 2019). Here, we found that α-synuclein was upregulated in DAergic-like neurons after MPP^+^ exposure, while pretreatment with HS significantly downregulated α-synuclein accumulation. Hence, the results from our cellular model of PD demonstrated that HS provided neuroprotection for DAergic-like neurons against damage and death induced by MPP^+^ through its stimulation of anti-apoptosis proteins, improvement of mitochondrial membrane potential, increase in TH synthesis, and suppression of α-synuclein synthesis.

In conclusion, we found that HS first exerted both neuroprotective and neurorestorative effects against MPTP-induced impairments in mouse model of PD, with higher efficacy in neuroprotection than neurorestoration for both DA neurons and fibers and behavioral improvement. Neuroprotective effect of HS might be due to its stimulatory actions on the upregulation of Bcl-2 and downregulation of cleaved caspase 3 protein expressions and improved mitochondria function by preventing mitochondrial membrane depolarization. Moreover, HS could stimulate the production of tyrosine hydroxylase (TH) and decreased the production of α-synuclein. Therefore, HS could be considered an attractive preventive and therapeutic agent for PD whose application in human PD needs further studies.

## Data Availability Statement

The original contributions presented in the study are included in the article/Supplementary Material, further inquiries can be directed to the corresponding author/s.

## Ethics Statement

The animal study was reviewed and approved by the Animal Care and Use Committee, Faculty of Science, Mahidol University (Approval number, MUSC58-017-332).

## Author Contributions

KN and KC designed the concept and coordinated this study. KN performed the experiments, analyzed, interpreted the data, and drafted the manuscript. MS supported the experiment of the animal PD mouse model. KC and PS performed critical revisions of the manuscript. All authors read and approved the final manuscript.

## Conflict of Interest

The authors declare that the research was conducted in the absence of any commercial or financial relationships that could be construed as a potential conflict of interest.

## Supplementary Material

Supplementary Figure 1 **(A)** H&E stating of kidney, spleen, liver, and heart on day 0 and day 14 after mice were injected with HS 10 mg/kg/day through intraperitoneal for 7 consecutive days and received HS 200 mg/kg/day through oral gavage for 7 consecutive days. **(B)** Changes in the body weight gain (g) of mice received HS 10 mg/kg/day through intraperitoneal for 7 consecutive days, received HS 200 mg/kg/day through oral gavage for 7 consecutive days compared with NSS group.
