# Supplementary figures and images for: Neuroprotective and Neurorestorative Effects of Holothuria scabra Extract in the MPTP/MPP+-Induced Mouse and Cellular Models of Parkinson’s Disease
Source: Front Neurosci. 2020 Dec 21;14:575459. doi: 10.3389/fnins.2020.575459 (PMC7779621; doi:10.3389/fnins.2020.575459)

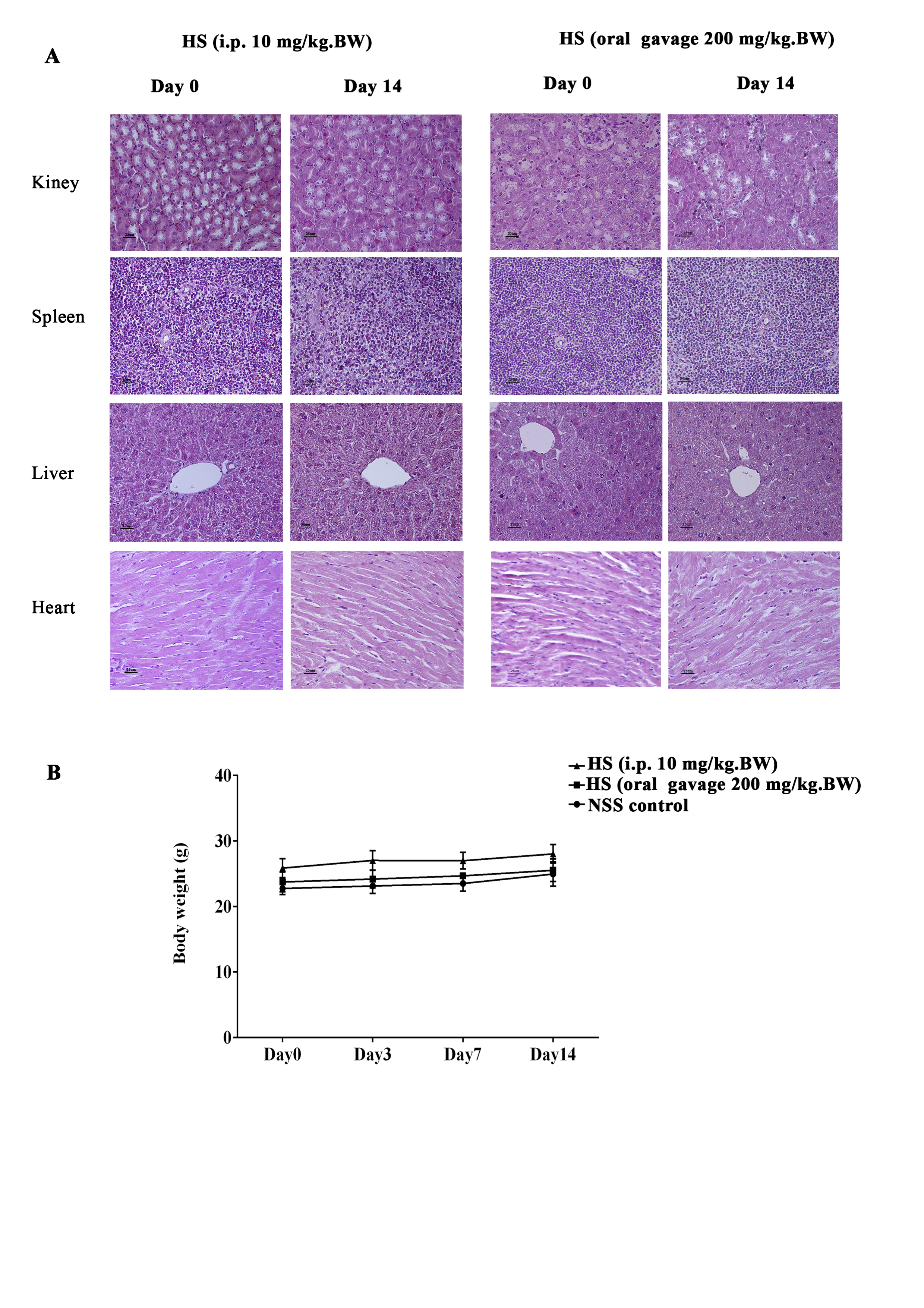

Supplement: Supplementary Figure 1 — (A) H&E stating of kidney, spleen, liver, and heart on day 0 and day 14 after mice were injected with HS 10 mg/kg/day through intraperitoneal for 7 consecutive days and received HS 200 mg/kg/day through oral gavage for 7 consecutive days. (B) Changes in the body weight gain (g) of mice received HS 10 mg/kg/day through intraperitoneal for 7 consecutive days, received HS 200 mg/kg/day through oral gavage for 7 consecutive days compared with NSS group. [file Image_1.JPEG]
